# Miliary breast cancer brain metastasis: a rare and aggressive form of central nervous system metastasis

**DOI:** 10.1038/s41416-020-1001-9

**Published:** 2020-08-04

**Authors:** Matthew N. Mills, Kamran A. Ahmed

**Affiliations:** Department of Radiation Oncology, H. Lee Moffitt Cancer Center and Research Institute, Tampa, FL 33612 USA

**Keywords:** Breast cancer, Metastasis

## Abstract

Although breast cancer brain metastasis is an increasingly common occurrence, relatively little is known about miliary brain metastases, a rare subtype that presents unique diagnostic and management challenges. The present study from Bashour et al. proposes the first objective diagnostic imaging criteria, enabling improved future study.

## Main

The development of brain metastases in patients with breast cancer has become an increasingly common occurrence due to advances in both imaging techniques and systemic therapy.^1^ The most common central nervous system (CNS) metastasis is typically within the grey–white matter junction of the brain parenchyma. A rare subset of brain metastases, termed miliary metastasis (MiM), has several unique characteristics, leading to poor treatment outcomes. First described by Madow and Alpers in 1951 as ‘carcinomatous encephalitis,’^2^ MiM describes numerous, nearly diffuse small foci of disease within the perivascular space, as opposed to the true brain parenchyma.^3,4^ Current knowledge of MiM is limited to case reports in primary lung adenocarcinoma,^4–7^ melanoma,^8^ small-cell gastric carcinoma^9^ and breast cancer^10^ presenting unique diagnostic challenges for this aggressive brain metastasis subtype. The rarity of this diagnosis and the lack of an objective imaging diagnostic criteria for MiM have both played a role in the limited understanding of this disease.

In the study by Bashour et al.,^11^ objective diagnostic criterion for MiM are proposed, including at least 20 lesions per image on at least two non-contiguous MRI images (or at least 10 lesions per image on at least two non-contiguous CT images), with bilateral lesions involving both the supratentorial and infratentorial brain compartments. Using these criteria, they identified 21 cases (3.8%) of MiM in a retrospective cohort of 546 patients with breast cancer brain metastasis. This appears to be in line with previously reported studies on the incidence of MiM.^2^ Leptomeningeal carcinoma was noted in 13 of these 21 patients. Interestingly, 11 patients had previously identified brain metastasis prior to the development of MiM. Fifteen of the 21 patients received whole-brain radiation therapy (WBRT), as the remaining patients had either previously received WBRT or proceeded with less aggressive treatment. While prognosis was poor for these patients, those with hormone receptor (HR)-positive/HER2-positive disease had a significantly longer overall survival (OS) (10.8 versus 3.7 months), a finding that is similar to studies of parenchymal brain metastasis.^12^ Representing the largest series of MiM in the literature, as well as the first proposed imaging-based diagnostic criteria, this study represents an important step in our understanding of this disease. Although further study is needed to validate the proposed diagnostic criteria for MiM, the current study establishes uniform diagnostic criteria for this rare subtype across studies. Without uniform radiological diagnostic criteria agreed upon by investigators, elucidation of the biology and treatment of this rare subtype would be impossible.

As evidenced by this series, MiM poses a diagnostic challenge. Patients with MiM have a wide range of presentations, often with poorly localised neurological deficits ranging from gradual to hyperacute in nature,^3,4,6,8,10^ and rarely can even remain asymptomatic.^5^ Diagnostic imaging can be unreliable in the detection of MiM; MRI of the brain may fail to show detectable metastases.^3^ In addition, as opposed to most common brain metastases, MiM often does not enhance with contrast and can lack the surrounding vasogenic oedema.^4^ Even when miliary brain lesions are detected by MRI, the appropriate treatment can be delayed, as miliary brain lesions represent a broad differential diagnosis, including infectious, inflammatory and nutritional aetiologies.^13^

Much of the current understanding of MiM is based on postmortem pathological examination. Ogawa et al.^3^ found that MiM is localised within the perivascular (Virchow–Robin) space with extension into the subpial space without invasion of the brain parenchyma, consistent with prior reports.^4^ Bugalho et al.^9^ reported the only known case of MiM from primary small-cell gastric carcinoma. Because prior studies of small-cell lung cancer noted MiM, Bugalho et al. theorised that similar cell types may share specific metastatic patterns. Interestingly, there is a potential correlation between MiM and epidermal growth factor receptor (EGFR)-mutated non-small-cell lung cancer (NSCLC), with multiple case reports.^4,6^ In a study including 543 patients with stage IV NSCLC at presentation, Hsu et al.^7^ found that the EGFR mutation-positive group, compared with the EGFR wild-type group, had a significantly higher risk of MiM (4.1% versus 0.5%) and miliary lung metastases (11.6% versus 3.3%). Although Bashour et al.^11^ did not note any correlations with MiM and specific breast cancer subtypes, larger future studies may reveal significant associations with subtype or genomic alterations.

As MiM represents a particularly poor prognosis, management is typically palliative in nature, with patients choosing between radiotherapy and supportive care. Stereotactic radiosurgery and hippocampal avoidance WBRT have been shown to offer good local control with an improved toxicity profile over conventional WBRT.^14,15^ However, due to the diffuse nature of MiM, with a higher risk for hippocampal involvement, standard WBRT is probably the most appropriate treatment. While unlikely to prolong survival, WBRT does have the potential to improve neurological symptoms.^6^

Leptomeningeal metastasis is understood to be a unique subset of CNS metastasis with exceedingly poor survival, with distinctive characteristics, prognosis and management.^16^ While the current understanding is limited, it is possible that MiM may represent a unique clinical entity with important distinctions. In the pursuit of a better understanding of the pathogenesis and potential treatment options of MiM, the objective imaging diagnostic criteria proposed by Bashour et al.^11^ are an important first step.

## Data Availability

Not applicable.
